# Glucagon Effects on ^3^H-Histamine Uptake by the Isolated Guinea-Pig Heart during Anaphylaxis

**DOI:** 10.1155/2014/782709

**Published:** 2014-05-11

**Authors:** Mirko Rosic, Oberdan Parodi, Vladimir Jakovljevic, Maja Colic, Vladimir Zivkovic, Vuk Jokovic, Suzana Pantovic

**Affiliations:** ^1^Faculty of Medical Sciences, Department of Physiology, University of Kragujevac, 69 Svetozara Markovica Street, 34 000 Kragujevac, Serbia; ^2^Research Center of Serbian Academy of Arts and Sciences and the University of Kragujevac, Kragujevac, Serbia; ^3^Clinical Physiology Institute, National Council of Research, Viale G. Moruzzi 1, 56124 Pisa, Italy

## Abstract

We estimated the influence of acute glucagon applications on ^3^H-histamine uptake by the isolated guinea-pig heart, during a single ^3^H-histamine passage through the coronary circulation, before and during anaphylaxis, and the influence of glucagon on level of histamine, NO, O_2_^−^, and H_2_O_2_ in the venous effluent during anaphylaxis. Before anaphylaxis, glucagon pretreatment does not change ^3^H-histamine Umax and the level of endogenous histamine. At the same time, in the presence of glucagon, ^3^H-histamine Unet is increased and backflux is decreased when compared to the corresponding values in the absence of glucagon. During anaphylaxis, in the presence of glucagon, the values of ^3^H-histamine Umax and Unet are significantly higher and backflux is significantly lower in the presence of glucagon when compared to the corresponding values in the absence of glucagon. The level of endogenous histamine during anaphylaxis in the presence of glucagon (6.9–7.38 × 10^−8^ **μ**M) is significantly lower than the histamine level in the absence of glucagon (10.35–10.45 × 10^−8^ **μ**M). Glucagon pretreatment leads to a significant increase in NO release (5.69 nmol/mL) in comparison with the period before glucagon administration (2.49 nmol/mL). Then, in the presence of glucagon, O_2_^−^ level fails to increase during anaphylaxis. Also, our results show no significant differences in H_2_O_2_ levels before, during, and after anaphylaxis in the presence of glucagon, but these values are significantly lower than the corresponding values in the absence of glucagon. In conclusion, our results show that glucagon increases NO release and prevents the increased release of free radicals during anaphylaxis, and decreases histamine level in the venous effluent during cardiac anaphylaxis, which may be a consequence of decreased histamine release and/or intensified histamine capturing by the heart during anaphylaxis.

## 1. Introduction

Anaphylaxis is a serious allergic reaction in which specific antigens provoke a sudden release of mast cell-derived and basophil-derived mediators of allergic phenomena including histamine, platelet-activating factor, leukotrienes, prostaglandins, tryptase, serotonin, cytokines, and nitric oxide (NO) [1–5]. In addition, mast cells produce reactive oxygen species (ROS), such as superoxide anion radical (O_2_^−^) and hydrogen peroxide (H_2_O_2_) [6], that are responsible for the increase of histamine release from the mast cells [7, 8]. Then, released histamine causes mast cell degranulation through stimulation of H_1_ receptors and leads to the further release of mast cell-derived mediators [7].

Immunologically released histamine plays a main role in genesis of most functional changes occurring in the anaphylaxis [3]. This biogenic amine not only exists within the mast cells and basophilic leukocytes, but it also could be released by endothelial cells, aggregating platelets, lymphocytes, and monocytes/macrophages as well as enterochromaffin-like cells and histamine neurons [9–11]. After release, histamine extracellular concentration is tightly regulated in order to terminate its effects via histamine receptors on target cells. Histamine clearance from the extracellular space occurs via plasma membrane transporters rather than through enzyme inactivation [12]. Although histamine plasma membrane transporters, organic cation transporter 3 (OCT3/EMT) [10, 12–15] and plasma membrane monoamine transporter (PMAT) [16, 17], have been found in the heart, regulation of cellular histamine uptake is still largely unknown. Histamine provokes various cellular functions by binding to four different G-protein-coupled receptors (H_1_, H_2_, H_3_, and H_4_) [18]. It causes both positive inotropic and positive chronotropic effects by stimulation of H_2_ receptors [19, 20]. The major arrhythmogenic actions of histamine are H_1_-receptor-mediated slowing of atrioventricular (AV) conduction and H_2_-receptor-mediated increases in sinus rate and ventricular automaticity [11, 21–23]. Previous studies have shown that histamine affects atherosclerosis progression through H_1_ receptor mechanisms [24]. Histamine enhances the expression of adhesion molecules in vascular endothelial cells, thereby augmenting leukocyte-endothelial cell interactions, an important event in atherogenesis [24]. Moreover, this biogenic amine suppresses hepatic LDL receptor expression and reduces plasma HDL cholesterol in rats [25]. This suggests that histamine may play an important role in lipoprotein metabolism, which may be related to its role in the development of atherosclerosis. Also, it has been shown that histamine increased smooth muscle cell proliferation and migration and is implicated in intimal thickening and atherogenesis [26]. Histamine's effects on coronary arteries are the result of multiple actions of this molecule on both smooth muscles and endothelial cells. Relaxation and constriction of coronary vessels have been widely reported as its effects. These effects are species specific and depend on dose of histamine, 1 diameter and initial vessel tone, as well as relative location within the coronary circulation [27]. Previous researches also showed that Kounis syndrome, as the concurrence of acute coronary syndromes, is associated with mast cell activation, such as allergies and hypersensitivity as well as anaphylactic or anaphylactoid insults [28]. In this respect, Kounis reported that any substances which protect mast cell surface and stabilize mast cell membrane could appear as a new therapeutic way capable of preventing acute coronary and acute cerebrovascular events.

Glucagon is an endogenous polypeptide hormone that exerts positive inotropic and chronotropic effects on the myocardial tissue [29, 30]. Cardiac effects of glucagon are considered to be resultant from stimulation of glucagon receptors associated with Gs protein, which cause adenylyl cyclase (AC) activation and the consequent increase of 3′,5′,-cyclic adenosine monophosphate (cAMP) production [31]. Stimulation of adenylyl cyclase leads to increase of cytosolic Ca^2+^ and might be involved in the inotropic effect of glucagon [32, 33]. In that way, it induces increase in contractility only in ventricle, but not in atrium, which appears to be a consequence of a lower glucagon receptor density in this tissue [29]. Andjelkovic and Zlokovic came into conclusion, a long time ago, that glucagon pretreatment decreased histamine release during anaphylaxis and thus caused beneficial effects in the heart during cardiac anaphylaxis [1]. They reported that the cardiac anaphylactic crisis was markedly reduced in the presence of glucagon and that antiarrhythmic action of glucagon in cardiac anaphylaxis involves inhibition of immunological histamine release, vasodilatation of the coronary vessels, increase in sinoatrial nodal automaticity, and enhancement of atrioventricular conduction velocity [1]. It is considered that inhibition of histamine release and vasodilatation probably play a major role in the mechanism of this antiarrhythmic activity [1]. In addition, it has been reported that glucagon influenced expression of vesicular monoamine transporter 2 (VMAT2) by which histamine is transported from the cytoplasm to storage vesicles of enterochromaffin-like cells [34]. The VMAT2 was also found to be expressed in mast cells of all human and monkey organs, megakaryocytes, platelets, basophil granulocytes, and cutaneous Langerhans cells [35]. However, there is *а* lack of data considering the possible effect of glucagon on histamine plasma membrane transporters (OCT3/EMT and PMAT) and thereby on histamine uptake from the extracellular space. The possible effect of glucagon on histamine release and/or on histamine uptake from the extracellular space could be relevant to cardiac anaphylaxis especially if it would decrease histamine release/downstream effects.

In anaphylaxis, increased NO production occurs, which is ubiquitous signaling molecule produced from L-arginine by nitric oxide synthase (NOS). However, the kind of nitric oxide synthase responsible for NO production during anaphylaxis is not clearly established. It is thought that increase of inducible NOS (iNOS) requires a longer period of time [36, 37]. On the other hand, Sade et al. [4] reported that NO level increased by activating iNOS, but not endothelial NOS, after allergen administration in the heart. In addition, immunocytochemical and Western blot analyses revealed that the expression of nNOS is most evident in cardiac myocytes [38]. NO is a free radical* per se* which at low doses plays a protective role against myocardial injury whereas high concentration of NO exerts highly toxic and harmful effects [39–42]. Also, NO may stabilize mast cells [43] and inhibit the release of mediators of allergic phenomena. NO production is elevated under glucagon treatment in rat hepatocyte culture [44].

In order to investigate glucagon effects in cardiac anaphylaxis, in this study we estimated the influence of acute glucagon application on ^3^H-histamine uptake, during a single ^3^H-histamine passage through the coronary circulation, before and during the anaphylaxis, as well as the influence of glucagon on level of histamine, NO, and oxidative stress mediators during anaphylaxis.

## 2. Material and Methods

All research procedures were carried out in accordance with European Council Directive (86/609/EEC) and principles of Good Laboratory Practice (2004/9/EC, 2004/10/EC) and approved by the Ethical Committee for the Welfare of Experimental Animals, Faculty of Medical Sciences, University of Kragujevac.

The histamine uptake by the isolated guinea-pig heart was measured using a rapid dual-isotope dilution method [45] and Langendorff's technique for the isolated heart with constant perfusion flow. This method allows the measurement of ^3^H-histamineuptake (test molecule) in relation to ^14^C-mannitol (as an extracellular reference molecule) during their passage through the coronary blood vessels [46–49]. The lower values of ^3^H-histamine recovery (i.e., histamine dilution profile curve) in relation to reference molecule reflect its uptake.

### 2.1. Isolated Heart Preparation

Guinea-pigs of male sex, body mass 400 ± 30 g, were sensitized by three intraperitoneal injections of 4 mg kg^−1^ ovalbumin during three consecutive days. Sensitization of the animals was confirmed using Schultz-Dale's gut assay [50]. Three weeks after the last injection, guinea-pigs were killed by cervical dislocation. The hearts were rapidly isolated and retrogradely perfused via the aorta, according to Langendorff's technique at a constant flow of 5 mL min^−1^ (g wet heart weight)^−1^ of the perfusion buffer containing (in mM): NaCl 118.1, KCl 4.7, MgSO_4_ 1.66, NaHCO_3_ 24.88, KH_2_PO_4_ 1.18, glucose 5.5, sodium pyruvate 20, CaCl_2_ × 2H_2_O 2.52 (Merk, Darmstadt, Germany). The perfusion buffer was continuously bubbled with 95% O_2_ and 5% CO_2_, with the pH adjusted to 7.4 at 37°C. Cardiac function, heart rate, and contractility as well as perfusion pressure were observed and registered using transducer of Langendorff apparatus (Experimetria Ltd., Budapest, Hungary).

### 2.2. Protocol A

#### 2.2.1. The Influence of Anaphylaxis on Histamine Uptake

Following 20–30 min equilibration with perfusion buffer, a 50 *μ*L bolus (control bolus), containing ^14^C-mannitol as an extracellular reference molecule and ^3^H-histamine as a test molecule, was injected into the heart via the aortic cannula. The ^3^H-histamine concentration was 0.22 nM L^−1^ (10 *μ*Ci) and the concentration of ^14^C-mannitol was 0.07 nM L^−1^ (4 *μ*Ci). The radioisotopes ^14^C-mannitol and ^3^H-histamine were purchased from the Biomedical Technologies Inc., USA. The first 10 samples (3 drops in each sample) of perfusion effluent were sequentially collected (collection time is 60–120 s). After a period of several minutes (usually 20–30 min) anaphylaxis of isolated heart was induced by a slow injection (during 20–30 s) of 1 mg mL^−1^ ovalbumin through the aortic cannula. During the first 60–90 s of anaphylaxis test bolus containing ^3^H-histamine and ^14^C-mannitol was injected into the perfusion system. Samples of perfusion effluent were sequentially collected in the same manner as described for the control bolus. All the samples were prepared for scintillation counting (Rackbeta, LKB-Wallac counter) by addition of 2 mL 98% ethanol and 2 mL scintillation fluid. The scintillation fluid contained 0.1 g of POPOP (1.4-bis(2-(5-phenyloxazolyl))benzene) and 4 g of PPO (2.5-diphenyloxazole) per litre of toluene (Sigma-Aldrich Chemie GmbH, Germany).

The radioactivity of each isotope in the sample of venous effluent (as a percentage of the injected dose) was plotted against the collection time, in order to obtain concentration-time curves, that is, dilution profiles for both test and reference tracer.

The ^3^H-histamine uptake* U *(*%*) is estimated from the dilution profiles using (1), where ^3^H-histamine (^3^H-his) and ^14^C-mannitol (^14^C-mann) represent radioactivity (in counts min^−1^) recovered in successive effluent samples:

(1) U(%)=(1−H  3-hisC  14-mann)×100

The maximum uptake (*U*_max⁡_) of ^3^H-histamine is the mean of the uptake values taken over the time period during which the uptake has reached a plateau.

*U*_net_ is the net uptake over the hole testing period and is calculated as

(2) Unet(%)=(1−Total-recovered  H  3-hisTotal-recovered  C  14-mann)×100.

The backflux (BF) of ^3^H-histamine from intracellular to extracellular space is calculated as

(3) $$\begin{array}{c} \text{BF}\left(\%\right)=\frac{U_{\max}-U_{\text{net}}}{U_{\max}}\times 100. \end{array}$$

In our study, calculated values of *U*_net_, *U*_max⁡_, and backflux (BF) by means of unidirectional paired tracer dilution method may, in fact, represent two events:

1. ^3^H-histamine binding to its membrane receptors which leads to lower ^3^H-histamine dilution profiles in respect to reference molecule and/or
2. cellular internalization of ^3^H-histamine (i.e., cellular uptake) which can be estimated by the BF.

Higher level of BF indicates lower cellular internalization of test molecule and* vice versa.* For instance, if the BF of test molecule is 60%, this indicates that 40% of this molecule stay “captured” by cellular internalization after total recovery of reference molecule.

### 2.3. Protocol B

#### 2.3.1. The Influence of Glucagon on Histamine Uptake by the Heart during Anaphylaxis

Following 20–30 min equilibration with perfusion buffer, a glucagon (Sigma-Aldrich Chemie GmbH, Germany) was continuously injected into the coronary circulation through the aortic cannula. Final glucagon concentration in perfusion buffer was 0.15 *μ*M L^−1^. Five minutes after the start of glucagon perfusion, a 50 *μ*L bolus was injected into the perfusion system containing ^3^H-histamine and ^14^C-mannitol at the same concentration as previously described in protocol A. The first 10 samples (3 drops in each sample) of perfusion effluent were sequentially collected. After a period of 20–30 min and five minutes after the start of glucagon perfusion, anaphylaxis was induced in a manner previously described (as for protocol A). During the first 60–90 s of anaphylaxis, bolus containing ^3^H-histamine and ^14^C-mannitol was injected into the perfusion system. Samples of perfusion effluent were sequentially collected, prepared for scintillation counting, and analyzed in the same manner as described above.

The inhibitions of *U*_max⁡_ and *U*_net_ in the presence of glucagon are calculated as

(4) $$\begin{array}{c} \text{Inhibition}\left(\%\right)=\frac{U_{\text{control}}-U_{\text{test}}}{U_{\text{control}}}\times 100, \end{array}$$

where *U*_control_ represents the ^3^H-histamine uptake by the heart in the absence of glucagon and *U*_test_ is the ^3^H-histamine uptake by the heart in the presence of glucagon.

#### 2.3.2. The Influence of Glucagon on Histamine Level during Anaphylaxis

To investigate the effect of glucagon on histamine level during anaphylaxis, histamine concentrations in the coronary venous effluents were measured before, during, and after ovalbumin administration in the absence as well as in the presence of glucagon. The histamine concentrations were assessed using Shore's fluorometric method [51].

#### 2.3.3. The Influence of Glucagon on NO and Oxidative Stress Parameters during Anaphylaxis

*Biochemical Assays*. In collected samples of the coronary venous effluent, nitrites NO_2_^−^, superoxide anion radical O_2_^−^, and hydrogen peroxide H_2_O_2_ were determined using the spectrophotometrical method (Specord S-600 Analytic Jena).

*Nitrite Determination*. Nitric oxide decomposes rapidly to form stable metabolite nitrite/nitrate products. Nitrite level (NO_2_^−^) was measured and used as an index of nitric oxide (NO) production by using Griess's reagent. A total of 0.5 mL of perfusate was precipitated with 200 *μ*L of 30% sulfosalicylic acid, vortexed for 30 min, and centrifuged at 3000 g. Equal volumes of the supernatant and Griess's reagent, containing 1% sulfanilamide in 5% phosphoric acid/0.1% naphtalene ethylenediamine-di hydrochloride, were added and incubated for 10 min in the dark and measured at 543 nmol/1. The nitrite levels were calculated by using sodium nitrite as the standard [52]. 

*Determination of Superoxide Anion Radical*. The level of superoxide anion radical (O_2_^−^) was measured by NBT (nitroblue tetrazolium) reaction in TRIS buffer with coronary venous effluent and measured at 530 nm. Krebs-Henseleit solution was used as a blank probe [53]. 

*Determination of Hydrogen Peroxide*. Measurement of hydrogen peroxide (H_2_O_2_) is based on oxidation of Phenol Red by hydrogen peroxide, in a reaction catalyzed by horseradish peroxidase (HRPO) [54]. 200 *μ*L of perfusate was precipitated with 800 *μ*L of fresh made Phenol Red solution (PRS) and then 10 *μ*L of (1 : 20) HRPO (made* ex tempore*) was added. For blank probe (instead of coronary venous effluent) adequate volume of Krebs-Henseleit solution was used. The level of H_2_O_2_ was measured at 610 nm.

All drugs used for determination of oxidative stress parameters were purchased from Sigma-Aldrich Chemie GmbH, Germany.

### 2.4. Statistical Analysis

One-way ANOVA and independent *t*-test with the significance threshold of *P* < 0.05 were used for statistical comparisons of data. All statistical calculations were done with the computer program SPSS, version 13.0.

## 3. Results

### 3.1. The Influence of Anaphylaxis on Histamine Uptake

In the first group of experimental animals (*n* = 8) we investigated dynamics of ^3^H-histamine uptake by the heart, during a single ^3^H-histamine passage through the coronary circulation, before and during anaphylaxis, by means of two-bolus injection containing ^3^H-histamine and ^14^C-mannitol as it is described in Section 2.

**Figure 1:**
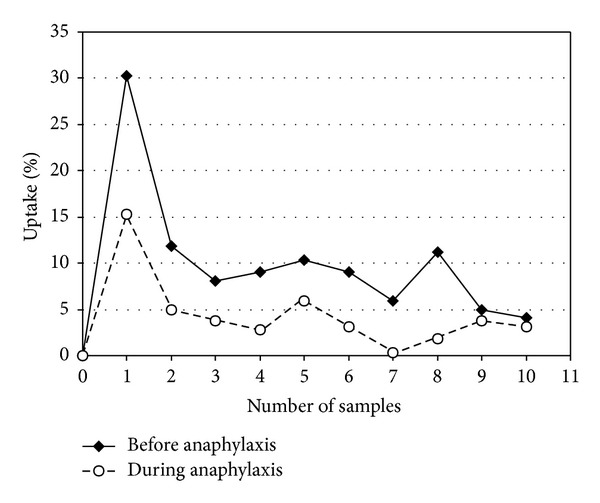
^3^H-histamine uptake by the heart before and during anaphylaxis (one representative experiment is shown).

The ^3^H-histamine uptakes before (control bolus) and during anaphylaxis (test bolus) were calculated using (1) (see Section 2) and shown in Figure 1. The maximum of ^3^H-histamine uptake (*U*_max⁡_) appears in the first sample (within 4–6 s) of the venous effluent after the injection of radiolabeled molecules. A lower profile of ^3^H-histamine uptake curve during anaphylaxis in relation to control curve indicates the inhibition of ^3^H-histamine uptake by the heart during the antigenic challenge (Figure 1).

The values of *U*_net_, *U*_max⁡_, and backflux (BF), before and during anaphylaxis, are represented in Table 1. All these parameters were calculated according to (2), (3), and (4) as described in Section 2. Our results indicate significant decrease (*P* < 0.05) of *U*_net_ and *U*_max⁡_ during anaphylaxis with unchanged backflux.

### 3.2. The Influence of Glucagon on Histamine Uptake by the Heart during Anaphylaxis

In the second group of experimental animals (*n* = 8) we investigated dynamics of ^3^H-histamine uptake by the heart, during a single ^3^H-histamine passage through the coronary circulation, before and during anaphylaxis, but, in this case, in the presence of glucagon.

**Figure 2:**
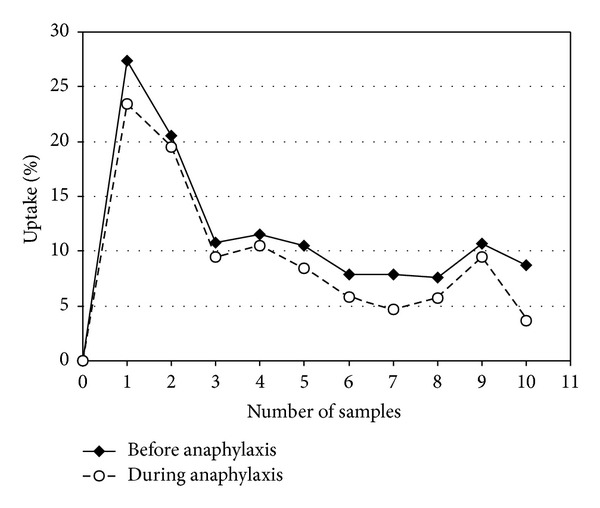
^3^H-histamine uptake by the heart before and during anaphylaxis in the presence of glucagon (one representative experiment is shown).

The ^3^H-histamine uptakes before (control bolus) and during anaphylaxis (test bolus), in the presence of glucagon, are presented in Figure 2. The maximum of ^3^H-histamine uptake (*U*_max⁡_) appears in the first sample (within 6–8 s) of the venous effluent after injection of radiolabeled molecules.

The values of *U*_net_, *U*_max⁡_, and backflux (BF), before and during anaphylaxis in the presence of glucagon, are represented in Table 1. All these parameters were calculated according to (2), (3), and (4) as described in Section 2. Our results indicate unchanged *U*_net_, *U*_max⁡_, and backflux during anaphylaxis (*P* > 0.05) in the presence of glucagon when compared to the corresponding values before anaphylaxis.

However, in comparison of these results to the corresponding values obtained in the absence of glucagon (represented in Table 1), our results suggest the following:

- the *U*_net_ before anaphylaxis in the presence of glucagon (17.34%) is significantly higher than in the absence of glucagon (12.4%);
- the *U*_net_ during anaphylaxis in the presence of glucagon (16.15%) is significantly higher than in the absence of glucagon (7.0%);
- the *U*_max⁡_ before anaphylaxis in the presence of glucagon (27.18%) is not significantly different than in the absence of glucagon (29.29%);
- the *U*_max⁡_ during anaphylaxis in the presence of glucagon (24.15%) is significantly higher than in the absence of glucagon (15.05%);
- the BF, before and during anaphylaxis in the presence of glucagon (36.38% and 33.24%, resp.), is significantly lower than in the absence of glucagon (57.6% and 53.72%, resp.).

The *U*_max⁡_ mostly represents the rapid initial binding of exogenously injected ^3^H-histamine to its membrane receptors/transporters. When a similar amount of endogenous histamine is present in the tissue, similar *U*_max⁡_ of exogenously injected ^3^H-histamine can be expected. This is exactly what happened in our research during glucagon pretreatment before anaphylaxis when *U*_max⁡_ was not changed (Table 1) because the level of endogenous histamine before anaphylaxis in the presence of glucagon was unchanged (Table 3). During anaphylaxis, ^3^H-histamine *U*_max⁡_ was significantly higher in the presence of glucagon (Table 1) when compared to the corresponding value in the absence of glucagon. At the same time, the level of endogenous histamine in effluent was lower in the presence of glucagon; see Tables 2 and 3. This means that glucagon during anaphylaxis reduced the level of endogenous histamine, which led to increase of exogenous^ 3^H-histamine binding to its membrane receptors/transporters of the heart cells and, consequently, to an increase in ^3^H-histamine *U*_max⁡_.

Then, our results indicate that glucagon pretreatment increases *U*_net_ of ^3^H-histamine and decreases its backflux (BF), before anaphylaxis. Decrease of the BF in the presence of glucagon indicates that glucagon intensifies histamine capturing by the cells in the heart, even before anaphylaxis. Glucagon-induced capturing of histamine by the heart cells consequently increases its total uptake (*U*_net_).

### 3.3. The Influence of Glucagon on Histamine Level during Anaphylaxis

To investigate the effect of glucagon on histamine level during anaphylaxis (*n* = 8), histamine concentrations in the coronary venous effluents were measured before, during, and after ovalbumin administration in the absence as well as in the presence of glucagon. Our results showed that significant increase of histamine release (*P* < 0.05) occurs during anaphylaxis (10.35–10.45 *·* 10^−8^ *μ*M) in the absence of glucagon (Table 2). This increase of histamine concentration achieved the maximum values during the first two minutes of the antigenic challenge and then decreased to the almost initial values within the next five minutes.

On the other hand there is no change in histamine concentration during glucagon perfusion before anaphylaxis compared to the period without glucagon (Table 3). During the first two minutes of anaphylactic reaction, an increase of histamine release occurred in the presence of glucagon (6.9–7.38 *·* 10^−8^ *μ*M). However, this increase of histamine level during anaphylaxis in the presence of glucagon was significantly lower (*P* < 0.05) when compared to the increase of histamine release during anaphylaxis in the absence of glucagon (Tables 2 and 3).

### 3.4. The Influence of Glucagon on NO and Oxidative Stress Parameters during Anaphylaxis

To investigate glucagon effects on the NO level as well as on the oxidative stress parameters O_2_^−^ and H_2_O_2_, in collected samples of venous effluent, concentrations of O_2_^−^, H_2_O_2_, and NO were measured both in the absence (*n* = 8; Table 2) and in the presence of glucagon (*n* = 8; Table 3), before, during, and after ovalbumin administration.

There are no statistically significant differences (*P* > 0.05) in NO levels before and during anaphylaxis, in the absence of glucagon (Table 2). On the other hand, there is a significant increase (*P* < 0.05) of NO release in the presence of glucagon (5.69 nmol/mL) compared to its level before glucagon perfusion (2.49 nmol/mL), as it is shown in Table 3. The increase of NO concentration achieved a peak value within 4-5 minutes of glucagon perfusion.

Our results indicate significant increase (*P* < 0.05) of O_2_^−^ concentration during the antigenic challenge (38.42 nmol/mL), in the absence of glucagon, when compared to the control values (26.64 nmol/mL) as it is shown in Table 2. Furthermore, in the presence of glucagon, administration of the ovalbumin (anaphylaxis) failed to increase the production of O_2_^−^ (Table 3).

In the presence of glucagon, our results showed no statistically significant differences in H_2_O_2_ level before, during, and after ovalbumin administration (Table 3; *P* = NS), but all these values are significantly lower (*P* < 0.05) when compared to the corresponding values in the absence of glucagon represented in Table 2. On the other hand, in the group without glucagon, significant increase of H_2_O_2_ concentration (3.28 nmol/mL) occurs during ovalbumin administration, as it is shown in Table 2 (*P* < 0.05). After initial increase in the first minute of ovalbumin administration, H_2_O_2 _  stayed at higher level throughout the whole testing period.

### 3.5. Effects of Glucagon on Cardiac and Coronary Functions

Cardiac function, heart rate, and contractility as well as coronary perfusion pressure were observed and registered as it is describe in Section 2. Glucagon pretreatment caused an increase in heart rate (34%) and this increase remains unchanged during anaphylaxis. On the other hand, glucagon pretreatment induced decrease in coronary perfusion pressure (18%) while the increase in contractility was not significant (*P* = NS). During anaphylaxis, coronary perfusion pressure was significantly increased in the absence of glucagon (32.4%; *P* < 0.05) while in the presence of glucagon it was increased by only 16.5% compared to the initial value before glucagon treatment. This means that the increase of coronary perfusion pressure that occurred during anaphylaxis was reduced in the presence of glucagon. Positive inotropic effect occurring in anaphylaxis was not significantly changed in the presence of glucagon.

## 4. Discussion

In this study we have shown that glucagon increased NO release and prevented the increased release of free radicals during anaphylaxis, and decreased histamine level in the venous effluent during cardiac anaphylaxis, which may be a consequence of decreased histamine release and/or intensified histamine capturing by the cells in the heart during anaphylaxis.

In the first part of this study, ^3^H-histamine uptake by the heart was investigated before and during anaphylaxis. We applied ^3^H-histamine concentration of 0.22 nM L^−1^ which was very low so it did not cause any significant change in the heart function. Our results showed that the maximum of ^3^H-histamine uptake (*U*_max⁡_) appeared in the first few drops of coronary venous effluent, that is, in the first 5–10 seconds after its injection into the coronary circulation, indicating rapid histamine uptake by the heart. During the anaphylactic reaction significant inhibition of ^3^H-histamine uptake occurred (Table 1). This inhibition refers to both *U*_net_ and *U*_max⁡_ and has been estimated to 43.89% and 48.51%, respectively. The backflux (BF) of ^3^H-histamine during anaphylaxis was not significantly different compared to the control value (Table 1). Inhibition of ^3^H-histamine uptake by the heart during anaphylactic reaction probably occurs as a result of histamine release from sensitized mast cells. It is well known that during anaphylactic reactions relatively large amounts of histamine release, especially during the first few minutes after antigen administration [1]. Histamine, released from sensitized mast cells (endogenous histamine), may bind to specific receptors and/or transporters and thereby may prevent uptake of exogenously injected ^3^H-histamine. In addition, we must not neglect the possible role of the other mediators of anaphylactic reactions in the inhibition of ^3^H-histamine uptake. Although even the *U*_max⁡_ and *U*_net_ were significantly decreased during anaphylaxis, the BF stayed unchanged. This suggests that the mechanisms underlying ^3^H-histamine capturing during anaphylaxis were still effective.

More than 20 years ago, we investigated ^3^H-histamine transport in the isolated hearts of nonsensitized guinea-pigs [47, 48]. The ^3^H-histamine uptake parameters of nonsensitized guinea-pig hearts, obtained a long time ago, did not show any significant differences compared to their levels of sensitized guinea-pig hearts before anaphylaxis in this study. This suggests that animal's sensitization itself does not affect histamine uptake.

The results presented in this study indicate that glucagon pretreatment increases *U*_net_ of ^3^H-histamine and decreases its BF, before anaphylaxis. Also, during anaphylaxis, in the presence of glucagon, *U*_max⁡_ and *U*_net_ were significantly higher and BF was significantly lower when compared to the corresponding values in the absence of glucagon (Table 1). At the same time, glucagon significantly decreases histamine concentration in coronary venous effluent during anaphylaxis, without affecting histamine level before it. This indicates a possible attenuation of histamine release from the mast cells during anaphylaxis in the presence of glucagon and can explain increased *U*_max⁡_ of exogenously injected ^3^H-histamine in this situation.

Beside the higher *U*_max⁡_ of exogenously injected ^3^H-histamine in the presence of glucagon, as the consequence of decreased release of endogenous histamine during anaphylaxis, it seems like glucagon has another effect reflected by the changes in *U*_net_ and especially BF of ^3^H-histamine. Namely, decrease of BF in the presence of glucagon indicates that glucagon intensifies histamine capturing even before anaphylaxis, potentially by activating the mechanisms underlying the cellular histamine uptake leading to the clearance of histamine in the local histamine-enriched environment. It is well known that expression of histamine-metabolizing enzymes such as diamine oxidase (DAO, histaminase) and histamine N-methyl transferase (HMT) in some tissues contribute to the clearance of the histamine in the local histamine-enriched environment. However, there is lack of data considering the possible effect of glucagon on histamine-metabolizing enzymes and thereby on histamine level. Another possible and more probable mechanism of histamine elimination is cellular uptake of histamine. In fact, plasma membrane transporters for reuptake of monoamines: organic cation transporters (OCT) 2 and 3 have the ability to transport histamine from extracellular space into cells [12–14]. The OCT3/EMT has a broad tissue distribution and is also found in the heart and vascular system [12–14]. Another plasma membrane monoamine transporter (PMAT) has recently been cloned and characterized [17] and has ability to transport histamine bidirectionally [16]. However, regulation of cellular histamine uptake is still not well known; even histamine uptake by mast cells has been documented near 50 years ago [55].

Before anaphylaxis, there is no significant difference in the *U*_max⁡_ obtained in the presence of glucagon and in the absence of glucagon as it is shown in Table 1. At the same time, there are no significant differences in the histamine levels in the venous effluents before anaphylaxis in the presence and in the absence of glucagon, as it is shown in Tables 2 and 3. The *U*_max⁡_ mostly represents the rapid initial binding of ^3^H-histamine to its membrane receptors and/or transporters. When a similar amount of endogenous histamine is present in the tissue, similar *U*_max⁡_ of exogenously injected ^3^H-histamine can be expected.

Taking into account all previously mentioned we postulate that glucagon decreases histamine release from cardiac mast cells during anaphylaxis and activates the mechanism(s) responsible for the clearance of histamine in the local histamine-enriched environment. These data are in accordance with the previous reports describing the influence of the glucagon on the inhibition of histamine release during anaphylaxis in the guinea-pigs isolated hearts and the protective effects of the glucagon pretreatments during cardiac anaphylaxis [1].

In the second part of this study, we investigated NO and parameters of the oxidative stress during anaphylaxis in the absence and in the presence of glucagon. It is well known that cardiac anaphylaxis is characterized by functional and biochemical changes of the hearts that are mainly attributed to the release of proinflammatory and vasoactive mediators including nitric oxide (NO) and reactive oxygen species (ROS) [2, 56, 57]. The ROS can participate as benevolent molecules in cells signaling processes. Also, an excessive increase in ROS production in the cardiovascular system in response to various stressors and in the failing heart can induce irreversible cellular damage and death [56]. The NO is a free radical* per se* but it can quench other free radicals, including the superoxide radicals, and thereby protect cells from damage [42, 58]. Also, NO may stabilize mast cells [43] and inhibit the release of mediators of allergic phenomena. Thus, low doses of NO appear to play a key protective role against myocardial injury whereas high concentration of NO exerts highly toxic and harmful effects [39–42].

Investigations of the NO level both before and during anaphylactic reaction in the presence and in the absence of glucagon clearly demonstrated the following (Tables 2 and 3):

- the NO level during glucagon perfusion was significantly elevated compared to the period before glucagon perfusion (Table 3);
- the NO level during anaphylactic reaction in the presence of glucagon (Table 3) was significantly higher compared to the corresponding NO level in the group without glucagon (Table 2), but there were no significant differences in NO levels between the periods before and during anaphylaxis in the presence of glucagon (Table 3);
- there are no statistically significant differences in NO levels between samples of coronary venous effluent collected throughout the testing period in the group without glucagon perfusion (Table 2).

These results are similar to the previous data reporting that the NO levels were elevated under glucagon treatment in the rat hepatocyte culture by activating inducible NOS [39]. In contrary to our results, Sade et al. found that NO levels increased during anaphylaxis by activating inducible NOS and thus elevating NO production within 15–30 minutes after allergen challenge in the heart [4].

From the present data, it is not possible to identify the source of NO released in the presence of glucagon. Previous researches revealed the existence of 3 distinct isoforms of nitric oxide synthase (NOS) that differ in activity and distributions in the tissues [4]. Constitutive NOS, neuronal NOS (nNOS) in the brain, and cardiac myocytes and endothelial NOS (eNOS) in blood vessels are tightly regulated in order to generate only small amounts of NO. On the other hand, inducible NOS (iNOS) is regulated mainly at the transcriptional level and produces large amount of NO. Since iNOS is regulated mainly at the transcriptional level, its activation depends on* de novo* synthesis of both RNA and protein and requires a longer period of at least 15–30 minutes [4]. This may be the reason explaining the same level of NO before and during anaphylaxis, since the collection time of venous effluent in our experiments was only 5-6 min (Table 2). Within that time initially increased histamine level in our investigation returns to its control value. The NO level in our study was increased as early as a few minutes (3–5 minutes) following the start of glucagon perfusion, implying iNOS is hardly to be responsible for its elevated level. In addition, according to other studies [59], glucagon failed to alter eNOS expression in endothelial cells implying eNOS is also hard to be responsible for NO elevated level in this study. On the other hand, immunocytochemical and Western blot analyses revealed that the expression of nNOS is most evident in cardiac myocytes [38]. Also, the effects of glucagon on cardiac myocardial cells are often correlated with adenylyl cyclase stimulation and cAMP-dependent phosphorylation of L-type Ca^2+^ channels [32], and since other studies [60, 61] have been clearly demonstrated positive correlation between intracellular increase of Ca^2+^ and nNOS in the myocardial cells, it can be postulated that glucagon may elevate NO level by influencing nNOS in cardiac myocytes.

Furthermore, it has been clearly demonstrated that elevated NO level might be the cause for stabilization of mast cells and consequently the inhibition of mast cell-released mediators of allergic phenomena such as histamine, nitric oxide, superoxide anion, and hydrogen peroxide [43, 62]. On that way glucagon may prevent histamine release from cardiac mast cells by increasing the NO production and thus stabilizing cardiac mast cells. This is in accordance with the results of our previous study [63] in which we investigated the influence of glucagon on ischemic vasodilatation of the isolated rat heart.

As expected, we noticed increased release of O_2_^−^ and H_2_O_2 _during ovalbumin application (anaphylaxis) (Table 2), which was in accordance with well-known role of free radicals in allergic reactions [64–66]. Moreover, O_2_^−^ can challenge mast cells to release the proinflammatory mediators, including histamine, thereby exaggerating the inflammatory response [67, 68]. Glucagon application prevents the increased release of O_2_^−^ and H_2_O_2_ during anaphylaxis in this study (Table 3). Taking into account all previously mentioned, we consider that glucagon can cause these effects in the following ways:

- by stabilization of mast cells via an increased level of NO and/or
- by reducing the level of histamine.

When guinea-pig isolated heart was perfused with glucagon we noticed increases in the heart rate while increases in myocardial contractility were not statistically significant (*P* < 0.05). These results confirm the previous reports describing the positive chronotropic and inotropic effects of glucagon on the myocardial tissue [1, 29, 30]. In addition, glucagon pretreatment caused a decrease in coronary perfusion pressure as a consequence of vasodilatation of the coronary vessels. It is thought that cardiac effects of glucagon are considered to be resultant from stimulation of glucagon receptors associated with Gs protein [31]. In our study we found that NO level during glucagon perfusion was significantly elevated compared to the period before perfusion (Table 3). This data points to the fact that coronary vasodilatation may be resultant of several factors which include not only the effects through specific glucagon receptors but also the effects of NO. Furthermore, glucagon-induced vasodilatation prevents higher increase in coronary perfusion pressure that occurs during anaphylaxis. In this study, the ECG is not registered. Previous research has shown [1] that glucagon has antiarrhythmic effect during anaphylaxis and that inhibition of histamine release and vasodilatation probably play a major role in the mechanism of this antiarrhythmic activity of glucagon.

## 5. Conclusion

Anaphylaxis is a serious allergic reaction in which specific antigens provoke a sudden release of mediators of allergic phenomena such as histamine, nitric oxide (NO), superoxide anion radical (O_2_^−^), and hydrogen peroxide (H_2_O_2_). It is thought that stabilization of cardiac mast cells appears to play a crucial role in the cardiac antianaphylactic reaction.

In this study we investigated the influence of acute glucagon applications on ^3^H-histamine uptake by the heart, before and during anaphylaxis, as well as the influence of glucagon on level of histamine, NO, and oxidative stress parameters in the venous effluent during anaphylaxis.

We showed that glucagon protective effects during cardiac anaphylaxis may be the result of

- decreased histamine level during cardiac anaphylaxis, which may be a consequence of decreased histamine release and/or intensified histamine capturing by the cells in the heart,
- increased NO release,
- prevented the increased release of free radicals.

Finally, even though the mechanism(s) of the beneficial action of glucagon on cardiac anaphylaxis is still unclear, the present study extends our knowledge and clearly indicates the key involvement of histamine, NO, and free radicals during this process.

## Figures and Tables

**Table 1:** Calculated values of *U*_net_,  *U*_max⁡_, and backflux (BF) before and during anaphylaxis in the absence and in the presence of glucagon as well as percentage of inhibition (% INH) of ^3^H-histamine uptake parameters during anaphylaxis.

	*U*_net_ (%)		*U*_max⁡_ (%)		BF (%)	
	Without glucagon	With glucagon	Without glucagon	With glucagon	Without glucagon	With glucagon
Before anaphylaxis	12.4 ± 1.23^∗†^	17.34 ± 2.1*	29.29 ± 1.37^†^	27.18 ± 1.41	57.6 ± 4.46*	36.38 ± 4.94*
During anaphylaxis	7.00 ± 1.24^∗†^	16.15 ± 1.66*	15.05 ± 1.69^∗†^	24.15 ± 1.49*	53.72 ± 4.0*	33.24 ± 3.63*
% INH	43.89 ± 5.26		48.51 ± 6.29			

Data are presented as mean ± SD, *n* = 8.

*Significantly different values (*P* < 0.05) between the period with and without glucagon.

^†^Significantly different values (*P* < 0.05) between the period before and during anaphylaxis.

**Table 2:** Obtained values of histamine, NO, O_2_^−^, and H_2_O_2_ in the samples of coronary venous effluent collected in the absence of glucagon: before ovalbumin application, during ovalbumin application (anaphylaxis), 1 min after ovalbumin application, and 5 min after ovalbumin application.

	Histamine (*μ*M)	NO (nmol/mL)	O_2_^−^ (nmol/mL)	H_2_O_2_ (nmol/mL)
Before ovalbumin application	4.8 ± 0.42 × 10^−8^	2.49 ± 0.12	26.64 ± 1.47	2.16 ± 0.19
During ovalbumin application (anaphylaxis)	10.35* ± 0.52 × 10^−8^	2.58 ± 0.10	38.42* ± 1.15	3.28* ± 0.14
One minute after ovalbumin application	10.45* ± 0.51 × 10^−8^	1.89 ± 0.16	36.85 ± 2.28	3.73 ± 0.25
Five minutes after ovalbumin application	5.83 ± 0.6 × 10^−8^	2.92 ± 0.42	37.44 ± 2.4	3.94 ± 0.45

Data are presented as mean ± SE, *n* = 8.

*Significantly different values (*P* < 0.05).

**Table 3:** Obtained values of histamine, NO, O_2_^−^, and H_2_O_2_ in the samples of coronary venous effluent collected: before glucagon perfusion, during glucagon perfusion (sample collected during 4-5 min of glucagon perfusion) but before ovalbumin application, during ovalbumin application, that is, in the first 60 s of anaphylaxis in the presence of glucagon, 1 min after ovalbumin application, in the presence of glucagon, and 5 min after ovalbumin application, in the absence of glucagon.

	Histamine (*μ*M)	NO (nmol/mL)	O_2_^−^ (nmol/mL)	H_2_O_2_ (nmol/mL)
Before glucagon perfusion	4.8 ± 0.42 × 10^−8^	2.49 ± 0.12	26.64 ± 1.47	2.16 ± 0.19

During glucagon perfusion and before ovalbumin application	4.98 ± 0.2 × 10^−8^	5.69* ± 0.3	21.08 ± 2.67	2.19 ± 0.26

During ovalbumin application (anaphylaxis) in the presence of glucagon	6.9* ± 0.25 × 10^−8^	4.77* ± 0.33	29.57 ± 1.36	1.74 ± 0.11

One minute after ovalbumin application in the presence of glucagon	7.38* ± 0.28 × 10^−8^	3.1 ± 0.19	30.58 ± 2.1	2.31 ± 0.12

Five minutes after ovalbumin application in the absence of glucagon	5.18 ± 0.2 × 10^−8^	4.7 ± 1.0	31.62 ± 2.8	2.71 ± 0.24

Data are presented as mean ± SE, *n* = 8.

*Significantly different values (*P* < 0.05).

## References

[1] Andjelkovic I, Zlokovic B (1982). Protective effects of glucagon during the anaphylactic response in guinea-pig isolated heart. *British Journal of Pharmacology*.

[2] Capurro N, Levi R (1975). The heart as a target organ in systemic allergic reactions. Comparison of cardiac anaphylaxis in vivo and in vitro. *Circulation Research*.

[3] Levi R, Kuye JO (1974). Pharmacological characterization of cardiac histamine receptors: sensitivity to H1 receptor antagonists. *European Journal of Pharmacology*.

[4] Sade K, Schwartz IF, Etkin S, Schwartzenberg S, Levo Y, Kivity S (2007). Expression of inducible nitric oxide synthase in a mouse model of anaphylaxis. *Journal of Investigational Allergology and Clinical Immunology*.

[5] Zavecz JH, Levi R (1977). Separation of primary and secondary cardiovascular events in systemic anaphylaxis. *Circulation Research*.

[6] Henderson WR, Kaliner M (1978). Immunologic and nonimmunologic generation of superoxide from mast cells and basophils. *Journal of Clinical Investigation*.

[7] Singh M, Saini HK (2003). Resident cardiac mast cells and ischemia-reperfusion injury. *Journal of Cardiovascular Pharmacology and Therapeutics*.

[8] Wolfreys K, Oliveira D (1997). Alterations in intracellular reactive oxygen species generation and redox potential modulate mast cell function. *European Journal of Immunology*.

[9] Beaven MA (2009). Our perception of the mast cell from Paul Ehrlich to now. *European Journal of Immunology*.

[10] Tanaka S, Deai K, Inagaki M, Ichikawa A (2003). Uptake of histamine by mouse peritoneal macrophages and a macrophage cell line, RAW264.7. *American Journal of Physiology-Cell Physiology*.

[11] Wolff AA, Levi R (1986). Histamine and cardiac arrhythmias. *Circulation Research*.

[12] Ogasawara M, Yamauchi K, Satoh Y (2006). Recent advances in molecular pharmacology of the histamine systems: organic cation transporters as a histamine transporter and histamine metabolism. *Journal of Pharmacological Sciences*.

[13] Gründemann D, Liebich G, Kiefer N, Köster S, Schömig E (1999). Selective substrates for non-neuronal monoamine transporters. *Molecular Pharmacology*.

[14] Zwart R, Verhaagh S, Buitelaar M, Popp-Snijders C, Barlow DP (2001). Impaired activity of the extraneuronal monoamine transporter system known as uptake-2 in Orct3/Slc22a3-deficient mice. *Molecular and Cellular Biology*.

[15] Inui K, Masuda S, Saito H (2000). Cellular and molecular aspects of drug transport in the kidney. *Kidney International*.

[16] Engel K, Zhou M, Wang J (2004). Identification and characterization of a novel monoamine transporter in the human brain. *Journal of Biological Chemistry*.

[17] Engel K, Wang J (2005). Interaction of organic cations with a newly identified plasma membrane monoamine transporter. *Molecular Pharmacology*.

[18] Morse KL, Behan J, Laz TM (2001). Cloning and characterization of a novel human histamine receptor. *Journal of Pharmacology and Experimental Therapeutics*.

[19] Hageman GR, Urthaler F, Isobe JH, James TN (1979). Chronotropic and dromotropic effects of histamine on the canine heart. *Chest*.

[20] Hough LB (2001). Genomics meets histamine receptors: new subtypes, new receptors. *Molecular Pharmacology*.

[21] Flynn SB, Gristwood RW, Owen DAA (1979). Differentiation of the roles of histamine H1- and H2-receptors in the mediation of the effects of histamine in the isolated working heart of the guinea-pig. *British Journal of Pharmacology*.

[22] Trzeciakowski JP, Levi R (1982). Reduction of ventricular fibrillation threshold by histamine: resolution into separate H1 and H2-mediated components. *Journal of Pharmacology and Experimental Therapeutics*.

[23] Wolff AA, Levi R (1988). Ventricular arrhythmias parallel cardiac histamine efflux after coronary artery occlusion in the dog. *Agents and Actions*.

[24] Li H, Burkhardt C, Heinrich U, Brausch I, Xia N, Förstermann U (2003). Histamine upregulates gene expression of endothelial nitric oxide synthase in human vascular endothelial cells. *Circulation*.

[25] Liao W, Rudling M, Angelin B (1997). Novel effects of histamine on lipoprotein metabolism: suppression of hepatic low density lipoprotein receptor expression and reduction of plasma high density lipoprotein cholesterol in the rat. *Endocrinology*.

[26] Lindstedt KA, Mäyränpää MI, Kovanen PT (2007). Mast cells in vulnerable atherosclerotic plaques—a view to a kill. *Journal of Cellular and Molecular Medicine*.

[27] Van de Voorde J, Brochez V, Vanheel B (1994). Heterogeneous effects of histamine on isolated rat coronary arteries. *European Journal of Pharmacology*.

[28] Kounis NG, Mazarakis A, Tsigkas G, Giannopoulos S, Goudevenos J (2011). Kounis syndrome: a new twist on an old disease. *Future Cardiology*.

[29] Gonzalez-Muñoz C, Nieto-Cerón S, Cabezas-Herrera J, Hernández-Cascales J (2008). Glucagon increases contractility in ventricle but not in atrium of the rat heart. *European Journal of Pharmacology*.

[30] Kaplan YC, Hocaoglu N, Oransay K, Kalkan S, Tuncok Y (2008). Effect of glucagon on amitriptyline-induced cardiovascular toxicity in rats. *Human and Experimental Toxicology*.

[31] White CM (1999). A review of potential cardiovascular uses of intravenous glucagon administration. *Journal of Clinical Pharmacology*.

[32] Mery PF, Brechler V, Pavoine C, Pecker F, Fischmeister R (1990). Glucagon stimulates the cardiac Ca2+ current by activation of adenylyl cyclase and inhibition of phosphodiesterase. *Nature*.

[33] Sistare FD, Picking RA, Haynes RC (1985). Sensitivity of the response of cytosolic calcium in Quin-2-loaded rat hepatocytes to glucagon, adenine nucleosides, and adenine nucleotides. *Journal of Biological Chemistry*.

[34] Prinz C, Zanner R, Gerhard M (1999). The mechanism of histamine secretion from gastric enterochromaffin-like cells. *American Journal of Physiology*.

[35] Anlauf M, Schäfer MK-H, Schwark T (2006). Vesicular monoamine transporter 2 (VMAT2) expression in hematopoietic cells and in patients with systemic mastocytosis. *Journal of Histochemistry and Cytochemistry*.

[36] Moncada S, Higgs A (1993). The L-arginine-nitric oxide pathway. *New England Journal of Medicine*.

[37] Nathan C (1997). Inducible nitric oxide synthase: what difference does it make?. *Journal of Clinical Investigation*.

[38] Kawahara K, Hachiro T, Yokokawa T, Nakajima T, Yamauchi Y, Nakayama Y (2006). Ischemia/reperfusion-induced death of cardiac myocytes: possible involvement of nitric oxide in the coordination of ATP supply and demand during ischemia. *Journal of Molecular and Cellular Cardiology*.

[39] Bertuglia S, Giusti A (2003). Microvascular oxygenation, oxidative stress, NO suppression and superoxide dismutase during postischemic reperfusion. *American Journal of Physiology-Heart and Circulatory Physiology*.

[40] Gourine AV, Gonon AT, Pernow J (2001). Involvement of nitric oxide in cardioprotective effect of endothelin receptor antagonist during ischemia-reperfusion. *American Journal of Physiology-Heart and Circulatory Physiology*.

[41] Jugdutt BI (2002). Nitric oxide and cardioprotection during ischemia-reperfusion. *Heart Failure Reviews*.

[42] Li XS, Uriuda Y, Wang QD, Nordlander R, Sjöquist P-O, Pernow J (1996). Role of L-arginine in preventing myocardial and endothelial injury following ischaemia/reperfusion in the rat isolated heart. *Acta Physiologica Scandinavica*.

[43] Parikh V, Singh M (2001). Possible role of nitric oxide and mast cells in endotoxin-induced cardioprotection. *Pharmacological Research*.

[44] Farghali H, Hodis J, Kutinová-Canová N, Potměšil P, Kmoníčková E, Zídek Z (2008). Glucose release as a response to glucagon in rat hepatocyte culture: involvement of NO signaling. *Physiological Research*.

[45] Yudilevich DL, Mann GE (1982). Unidirectional uptake of substrates at the blood site of secretory epithelia: stomach, salivary gland, pancreas. *Federation Proceedings*.

[46] Kostic MM, Rosic GL, Segal MB, Rosic MA (1995). Biphasic L-arginine uptake by the isolated guinea-pig heart. *Experimental Physiology*.

[47] Rosic M, Andjelkovic I, Zlokovic B (1987). Characterization of ^3^H histamine transport at sarcolemal membrane of the isolated perfused guinea-pig heart in the presence of glucagon and H1 and H2 receptor antagonists. *Biomedical and Biochemical Acta*.

[48] Rosic M, Andjelkovic I, Zlokovic B (1988). Effects of glucagon and H1 and H2 receptor antagonists on ^3^H histamine transport at sarcolemmal membrane of the isolated perfused guinea pig heart. *Yugoslav Physiological and Pharmacological Acta*.

[49] Rosic MA, Pantovic SB, Lucic AP, Ribarac-Stepic N, Andjelkovic IZ (2001). Kinetics of thyroxine (T_4_) and triiodothyronine (T_3_) transport in the isolated rat heart. *Experimental Physiology*.

[50] Dale HH, Hartley P (1916). Anaphylaxis to the separated proteins of horse-serum. *Biochemical Journal*.

[51] Shore PA, Burkhalter A, Cohn VH (1959). A method for the fluorometric assay of histamine in tissues. *The Journal of Pharmacology and Experimental Therapeutics*.

[52] Green LC, Wagner DA, Glogowski J (1982). Analysis of nitrate, nitrite, and [15N]nitrate in biological fluids. *Analytical Biochemistry*.

[53] Auclair C, Voisin E, Greenwald RA (1985). Nitroblue tetrazolium reduction. *CRC Handbook of Methods For Oxygen Radical Research*.

[54] Pick E, Keisari Y (1980). A simple colorimetric method for the measurement of hydrogen peroxide produced by cells in culture. *Journal of Immunological Methods*.

[55] Cabut M, Haegermark Ö (1966). Uptake, storage and release of histamine by rat peritoneal mast cells in vitro. *Acta Physiologica Scandinavica*.

[56] Giordano FJ (2005). Oxygen, oxidative stress, hypoxia, and heart failure. *Journal of Clinical Investigation*.

[57] Masini E, Zagli G, Ndisang JF, Solazzo M, Mannaioni PF, Bani D (2002). Protective effect of relaxin in cardiac anaphylaxis: involvement of the nitric oxide pathway. *British Journal of Pharmacology*.

[58] Rubanyi GM, Ho EH, Cantor EH, Lumma WC, Botelho LHP (1991). Cytoprotective function of nitric oxide: inactivation of superoxide radicals produced by human leukocytes. *Biochemical and Biophysical Research Communications*.

[59] Ding Y, Vaziri ND, Coulson R, Kamanna VS, Roh DD (2000). Effects of stimulated hyperglycemia, insulin, and glucagon on endothelial nitric oxide synthase expression. *American Journal of Physiology-Endocrinology and Metabolism*.

[60] Luo CX, Zhu DY (2011). Research progress on neurobiology of neuronal nitric oxide synthase. *Neuroscience Bulletin*.

[61] Sears CE, Bryant SM, Ashley EA (2003). Cardiac neuronal nitric oxide synthase isoform regulates myocardial contraction and calcium handling. *Circulation Research*.

[62] Masini E, Gambassi F, di Bello MG, Mugnai L, Raspanti S, Mannaioni PF (1994). Nitric oxide modulates cardiac and mast cell anaphylaxis. *Agents and Actions*.

[63] Rosic M, Pantovic S, Rosic G (2010). Glucagon effects on ischemic vasodilatation in the isolated rat heart. *Journal of Biomedicine and Biotechnology*.

[64] Bowler RP, Crapo JD (2002). Oxidative stress in allergic respiratory diseases. *The Journal of Allergy and Clinical Immunology*.

[65] Calhoun WJ, Reed HE, Moest DR, Stevens CA (1992). Enhanced superoxide production by alveolar macrophages and air-space cells airway inflammation and alveolar macrophage density changes after segmental antigen bronchoprovocation in allergic subjects. *The American Review of Respiratory Disease*.

[66] Wu W, Samoszuk MK, Comhair SA (2000). Eosinophils generate brominating oxidants in allergen-induced asthma. *The Journal of Clinical Investigation*.

[67] Brightling CE, Bradding P, Symon FA, Holgate ST, Wardlaw AJ, Pavord ID (2002). Mast-cell infiltration of airway smooth muscle in asthma. *The New England Journal of Medicine*.

[68] Williams CM, Galli SJ (2000). Mast cells can amplify airway reactivity and features of chronic inflammation in an asthma model in mice. *The Journal of Experimental Medicine*.

